# The Role of Fibrocytes in Sickle Cell Lung Disease

**DOI:** 10.1371/journal.pone.0033702

**Published:** 2012-03-19

**Authors:** Joshua J. Field, Marie D. Burdick, Michael R. DeBaun, Brett A. Strieter, Ling Liu, Borna Mehrad, C. Edward Rose, Joel Linden, Robert M. Strieter

**Affiliations:** 1 Department of Medicine, Division of Pulmonary and Critical Care Medicine, The University of Virginia School of Medicine, Charlottesville, Virginia, United States of America; 2 Blood Center of Wisconsin, Blood Research Institute, Milwaukee, Wisconsin, United States of America; 3 Departments of Pediatrics and Medicine, Division of Hematology and Oncology, Vanderbilt University School of Medicine, Nashville, Tennessee, United States of America; 4 La Jolla Institute for Allergy & Immunology, La Jolla, California, United States of America; Johns Hopkins School of Medicine, United States of America

## Abstract

**Background:**

Interstitial lung disease is a frequent complication in sickle cell disease and is characterized by vascular remodeling and interstitial fibrosis. Bone marrow-derived fibrocytes have been shown to contribute to the pathogenesis of other interstitial lung diseases. The goal of this study was to define the contribution of fibrocytes to the pathogenesis of sickle cell lung disease.

**Methodology/Principal Findings:**

Fibrocytes were quantified and characterized in subjects with sickle cell disease or healthy controls, and in a model of sickle cell disease, the NY1DD mouse. The role of the chemokine ligand CXCL12 in trafficking of fibrocytes and phenotype of lung disease was examined in the animal model. We found elevated concentration of activated fibrocytes in the peripheral blood of subjects with sickle cell disease, which increased further during vaso-occlusive crises. There was a similar elevations in the numbers and activation phenotype of fibrocytes in the bone marrow, blood, and lungs of the NY1DD mouse, both at baseline and under conditions of hypoxia/re-oxygenation. In both subjects with sickle cell disease and the mouse model, fibrocytes expressed a hierarchy of chemokine receptors, with CXCR4 expressed on most fibrocytes, and CCR2 and CCR7 expressed on a smaller subset of cells. Depletion of the CXCR4 ligand, CXCL12, in the mouse model resulted in a marked reduction of fibrocyte trafficking into the lungs, reduced lung collagen content and improved lung compliance and histology.

**Conclusions:**

These data support the notion that activated fibrocytes play a significant role in the pathogenesis of sickle cell lung disease.

## Introduction

Sickle cell disease (SCD) is the most common monogenic inherited disorder in African-Americans [1]. The two most common complications of SCD, vaso-occlusive pain crises (VOC) and acute chest syndrome (ACS), are the major risk factors for the development of interstitial lung disease (ILD) [2], a risk for morbidity and mortality in patients with SCD [2], [3]. ILD is characterized by parenchymal and vascular remodeling among patients with SCD and has been associated with the development of pulmonary hypertension and, potentially through this mechanism, with increased mortality [2], [4]. Although most studies of SCLD have focused on vascular remodeling and pulmonary hypertension [5], patients with SCD have long been recognized to develop a progressive restrictive ventilatory defect and interstitial markings on chest radiographs [2], [6], [7]. While many adults with SCD do not exhibit all features of SCLD, a restrictive pattern on pulmonary function tests is the most consistent clinical feature; for example, 74% of adults in a large prospective cohort study SCD had restrictive lung disease [6]. These studies underscore the need to better understand the pathogenesis of ILD in patients with SCD.

Fibrotic lung diseases are associated with dysregulated repair in response to persistent or recurrent injury, leading to loss of alveolar-capillary basement membrane integrity and remodelling of the lung airpace, interstitial and vascular compartments [8], [9], [10], [11], [12]. The fibroblasts involved in this process are known to be derived, in part, from proliferation of resident lung fibroblasts. More recent evidence has implicated a bone marrow-derived circulating mesenchymal progenitor cell, the fibrocyte, in lung fibrosis and has changed the perspective of lung repair [13], [14], [15]. Fibrocytes express the hematopoietic stem cell antigen CD34, the common leukocyte marker CD45, the myeloid markers CD11b and CD13, and fibroblast markers vimentin, collagen I, collagen III and fibronectin, as well as several chemokine receptors [16], [17], [18]. Only a subset of circulating fibrocytes expresses CD34, and the expression of both CD34 and CD45 on fibrocytes decreases when the cells are cultured or after they enter tissue. Since there is no single marker unique for the identification of fibrocytes, the co-expression of collagen production and CD34 or CD45 has been commonly used to identify these cells [16], [17], [18]. Fibrocytes are important in mediating pulmonary fibrosis in murine models of lung injury [7], [13], [19], [20], [21], [22] and are elevated in the blood and lungs of patients with idiopathic pulmonary fibrosis (IPF) [23], [24], [25]. Moreover, the number of circulating fibrocytes is a predictor of prognosis in patients with IPF [25]. Given the role of fibrocytes in the pathogenesis of lung remodelling in other diseases, we postulated that these cells are important in the development of ILD in patients with SCD.

## Methods

Peripheral blood samples from adult patients with SCD or race-matched controls and bone marrow, blood and lungs of NY1DD mice or congenic C57BL/6 mice were processed for quantification and characterization of fibrocytes, as previously described [19], [23] (See the online supplement for additional details of methods used). In some experiments, animals were exposed to hypoxia-reoxygenation (3 h at 8% oxygen/92% nitrogen followed by 4 h at 21% oxygen/79% nitrogen) before tissue harvest. CXCL12 was neutralized in vivo as previously described [13], [26]. Lung mechanics of animals were assessed by FlexiVent using Quasi-static pressure-volume (PV) curve to calculate total lung compliance and elastance, and forced oscillatory measurements to measure frequency dependence of parenchymal tissue impedance and parenchymal tissue elastance according to manufacturer's instructions. Plasma samples were analyzed for cytokines by Luminex multiplex protein analysis as previously described [13]. Lung collagen content was quantified using the Sircol assays (Biocolor Ltd., Belfast, United Kingdom) according to the manufacturer's instructions [27] and using morphometric analysis of histological samples after picrosirius red staining [28]. To quantify picrosirius red stains, lungs were inflated, fixed and sectioned; photomicrographs were then obtained from 10 randomly selected medium power fields from the lung periphery in each slide. By obtaining images from the lung periphery only, we excluded fields that included large blood vessels or airways. In human subjects, fibrocyte concentrations were not distributed normally and were compared using the Mann-Whitney U test;. Subject demographics and SCD characteristics were analyzed with Chi-Square tests for categorical variables and either Student's t or Mann-Whitney U tests for continuous variables that were normally or non-normally distributed, respectively. Mouse fibrocyte data were normally distributed and were compared using Student's t test. Paired measurements were compared using Wilcoxon matched-pairs signed-ranks test. Probability values were considered statistically significant if they were less than 0.05. Data analysis was performed in SAS version 9.1 (SAS Institute, Cary, NC).

The Institutional Review Board (IRB) at Washington University and the University of Virginia approved all human protocols and written informed consent was obtained in accordance with the Declaration of Helsinki. IRB approval numbers are PRO00014330 and 15598. The animal studies were carried out in accordance with the recommendations in the Guide for the Care and Use of Laboratory Animals of the National Institutes of Health and were approved by the Committee on the Ethics of Animal Experiments of the University of Virginia (Permit Number: 3549).

Refer to Supplemental Information S1 for detailed methods.

## Results

### Fibrocytes are markedly elevated and activated in the circulation of patients with SCD at baseline

Since previous studies had demonstrated that elevated fibrocytes can be found in the circulation of patients with ILD [23], [25], we wanted to determine whether fibrocytes were present in patients with SCD under similar conditions (i.e., routine clinic visit with no clinical evidence of VOC). From a cohort of patients with SCD at Washington University in St. Louis, samples from 114 unique SCD patients along with 19 African American controls were collected and shipped overnight to the laboratory at UVA (RMS). Mean age was 34±12 years for the SCD cases and 41±4 years for the controls. Fifty-four percent of the SCD subjects were female, whereas 88% of the control participants were female. Among the SCD cases, 73% percent had HbSS or HbSbeta-thalassemia^0^; the remainder had HbSC and HbSbeta-thalassemia^+^. Nineteen percent of SCD subjects required home oxygen either continuously or at night. When subjects with more and less severe SCD phenotypes were compared (HbSS/HbSβ-thalassemia^0^ vs. HbSC/HbSβ-thalassemia^+^), there were no differences in circulating fibrocyte levels (Table 1).

**Table 1:** Baseline demographics, SCD characteristics and fibrocyte levels in SCD subjects with severe SCD phenotypes (HbSS/Sβ-thalassemia^0^) compared to milder SCD phenotypes (HbSC/Sβ-thalassemia^+^).

	SCD cohort n = 114	HbSS/Sβthal^0^n = 82	HbSC/Sβthal^+^n = 32	*P* value
**Demographics**				
Age, median (IQR)	30.9 (17)	28.5 (14)	39.3 (21)	**0.03**
Gender, % male	46	47	44	0.28
**SCD characteristics**				
Hemogloblin (g/dL), mean (SD)	9.1 (1.8)	8.5 (1.4)	10.5 (1.8)	**<0.001**
WBC (cells/µL), mean (SD)	11.2 (3.3)	11.7 (3.2)	9.8 (3.1)	**0.004**
LDH (IU/L), median (IQR)	311 (194)	360 (231)	244 (102)	**<0.001**
Hydroxyurea, %	33	44	6	**<0.001**
Supplemental oxygen, %	19	26	3	**0.006**
**Fibrocytes/ml×10^5^, median (IQR)**				
CD45+Col1+	3.47 (6.78)	3.58 (6.90)	2.63 (6.28)	0.49
CD45+Col1+CXCR4+	2.25 (6.15)	2.30 (6.54)	2.05 (6.18)	0.71
CD45+Col1+CCR2+	0.68 (1.54)	0.66 (1.67)	0.74 (1.58)	0.98
CD45+Col1+CCR7+	0.45 (1.46)	0.45 (1.55)	0.43 (1.14)	0.68
CD45+Col1+αSMA+	0.58 (1.81)	0.66 (1.90)	0.56 (1.58)	0.93
CD45+Col1+pSMAD2/3+	1.05 (3.56)	1.05 (3.75)	1.02 (3.51)	0.62

IQR, inter-quartile range; LDH, lactate dehydrogenase; SCD, sickle cell disease; SD, standard deviation; WBC, white blood cell.

**Figure 1:**
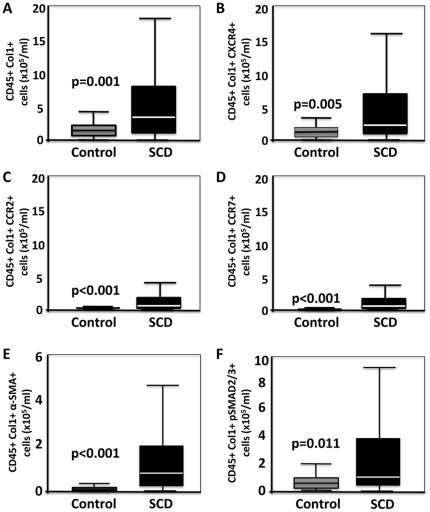
Fibrocytes are markedly elevated and activated in the circulation of patients with SCD at baseline compared to healthy African American controls. A) fibrocytes defined as CD45+Col1+ cells were elevated in number in the circulation of patients with SCD, as compared to control subjects. B–D) demonstrates that fibrocytes in the circulation of patients with SCD, as compared to control subjects express a chemokine receptor hierarchy (i.e., CXCR4+>CCR2+>CCR7+). E–F) demonstrates that fibrocytes in the circulation of patients with SCD, as compared to control subjects represent an activated phenotype of αSMA+ and pSmad2/3+ cells, respectively, compatible with fibrocytes pre-systemically activated by TGF-b.

We assessed de novo the circulating levels of fibrocytes (CD45+Col1+ cells) in these patients using quantitative FACS analysis as previously described [23], [25]. We chose to first gate on CD45+ rather than CD34+ cells, because we have historically found that the number of CD34+Col1+ fibrocytes in the circulation is an order of magnitude lower than CD45+Col1+ fibrocytes (data not shown). Fibrocyte levels were significantly higher in SCD cases compared to controls (median: 3.47×10^5^ cells/ml vs. 1.49×10^5^ cells/ml; p = 0.001) (Figure 1A). Further analysis of SCD cases demonstrated a hierarchy of chemokine receptor expression (CXCR4≫CCR2>CCR7) (Figure 1B, 1C, and 1D). While tissue myofibroblasts can arise from cells other than fibroytes, prior literature indicated that in both in vitro studies and animal models, fibrocytes represent a progenitor cell that can differentiate into αSMA-expressing myofibroblast-like cells [13], [29], [30] and human lungs contain fibrocytes expressing the myofibrobast marker αSMA [24]. We therefore also determined whether the elevation in circulating fibrocytes in the SCD patients was associated with increased numbers of αSMA+ fibrocytes in circulation by quantitative FACS analysis as previously described [23], [25], [30]. SCD patients had a higher number of αSMA+ fibrocytes compared to controls (Figure 1E). We previously determined that TGF-b stimulation of fibrocytes via activation of receptor Smads (Smad2/3) is critical to initiate signal transduction and induction of αSMA in these cells [29]. On this basis, we developed a strategy in our laboratory to detect activated receptor Smads (pSmad2/3) by quantitative FACS analysis, which we confirmed by using Western Blot analysis of cells stimulated with TGF-b. Fibrocytes in circulation of SCD patients were subsequently analyzed for activated receptor Smads (pSmad2/3) and were found to be significantly higher than controls (Figure 1F).

### Fibrocytes increase significantly over baseline level in patients with SCD under conditions of VOC

**Figure 2:**
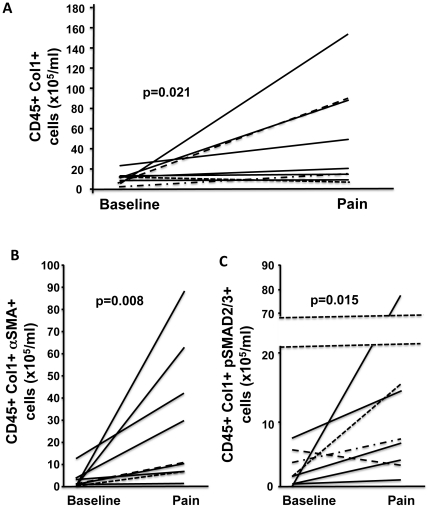
Fibrocytes increase significantly over baseline level in patients with SCD under conditions of VOC. A) Fibrocytes defined as CD45+Col1+ cells in nine SCD patients were markedly increased from baseline during an episode of VOC. B–C) demonstrates that the fibrocytes in the circulation of SCD patients undergoing a VOC represent an activated phenotype of αSMA+ and pSmad2/3+ cells, respectively, compatible with fibrocytes systemically pre-activated by TGF-b.

Blood from 9 SCD patients were analyzed at baseline and at time of admission to the hospital for VOC. Average age in this subset of patients was 35±9 years. Six subjects were female and 7 had HbSS/Sβ-thalassemia^0^. Notably, there were no significant differences in age, gender, SCD genotype or baseline fibrocyte levels between those SCD subjects with and without paired measurements (data not shown). During a vaso-occlusive episode compared to baseline, there were significant increases the number of circulating fibrocytes (p = 0.021), αSMA+ fibrocytes (p = 0.008), and pSmad2/3+ fibrocytes (0.015) (Figure 2A–C).

### NY1DD SCD mice demonstrated increased deposition of extracellular matrix in their lungs under baseline conditions

**Figure 3:**
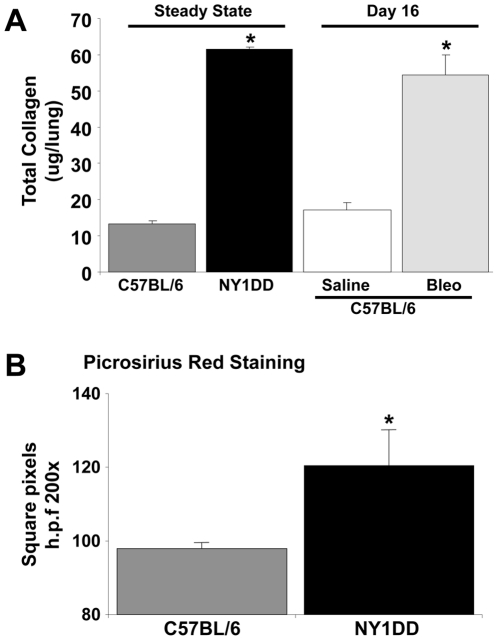
NY1DD SCD mice demonstrated increased deposition of extracellular matrix in their lungs under baseline conditions. A) NY1DD SCD mice, as compared to appropriate strain (C57Bl/6) and age-matched mice, demonstrate elevated levels of total collagen in their lungs under baseline (normoxia) conditions. The levels of total collagen in the lungs of NY1DD mice at baseline are equivalent to C57Bl/6 mice exposed to the pulmonary fibrotic agent, bleomycin, at day 16. B) NY1DD mice, as compared to appropriate strain and age-matched mice demonstrate elevated levels of collagen, as assessed by morphometric analysis of picrosirius red staining of lung tissue. N = six mice in each group. * p<0.05.

To more fully elucidate the role of fibrocytes in the development of ILD among patients with SCD, we next focused our studies on a murine model of SCD [31]. Prior studies have shown that significant injury from microvascular occlusion occurs during baseline conditions in the NY1DD mouse, including lung injury [31]. Since the NY1DD mice displayed evidence for marked lung injury under baseline conditions, we sought evidence of pulmonary fibroproliferation. Using the Sircol assay as previously described [13], [32] to measure total soluble collagen in the lungs of NY1DD mice at 6 to 8 weeks; we determined that these mice have increased levels of soluble collagen in their lungs, as compared to controls (Figure 3A). These findings were confirmed by morphometric analysis of the lungs by the collagen-specific dye picrosirius red (Figure 3B). To put into context the levels of soluble collagen in the lungs of these SCD mice with other mouse models of pulmonary fibrosis, we compared these lung soluble collagen levels to C57BL/6 mice that had been exposed to bleomycin for 16 days to produce pulmonary fibrosis [32], [33]. Levels of soluble collagen in bleomycin-exposed mice were similar to the baseline levels of soluble collagen in the lungs of NY1DD mice (Figure 3A).

### NY1DD SCD mice displayed increased numbers of fibrocytes in their bone marrow, circulation, and lungs under baseline conditions

**Figure 4:**
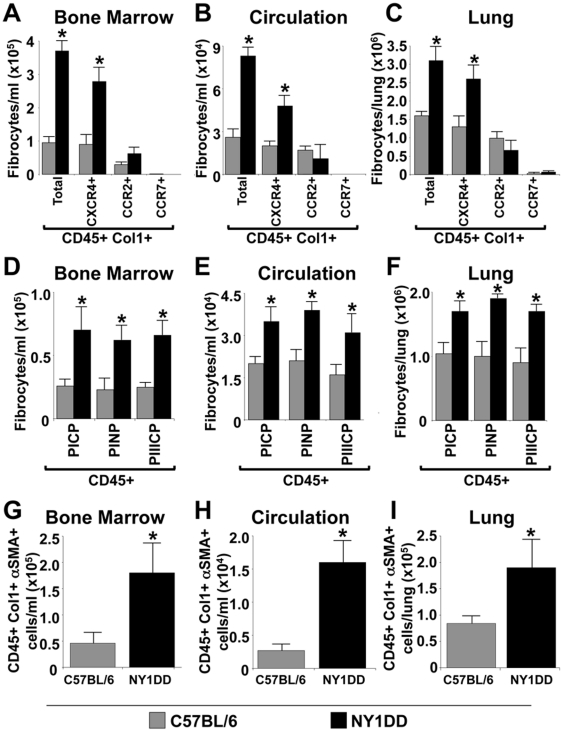
NY1DD SCD mice displayed increased numbers of fibrocytes in their bone marrow, circulation, and lungs under baseline conditions. A–C) demonstrates that fibrocytes are elevated in the bone marrow, circulation, and lungs of NY1DD SCD mice, as compared to strain and age-matched control mice. In addition, A–C) demonstrates that fibrocytes in the bone marrow, circulation, and lungs of NY1DD mice, as compared to strain and age-matched mice express a chemokine receptor hierarchy (i.e., CXCR4+≫CCR2+>CCR7+). D–F) demonstrates that elevated fibrocytes in the bone marrow, circulation, and lungs of NY1DD mice, as compared to appropriate strain and age-match mice express pro-collagens I and III (pro-collagen type I N and C-terminus = PINP and PICP, respectively; pro-collagen type III C-terminus = PIIICP). G–I) NY1DD SCD mice displayed increased numbers of fibrocytes (CD45+Col1+ cells) in their bone marrow, circulation, and lungs under baseline conditions that represent an activated phenotype (αSMA+ cells) compared to appropriate strain and age-match mice. N = six mice in each group. * p<0.05.

To determine whether the pathogenesis of increased fibroproliferation in the lungs of the NY1DD mice was due to the presence of fibrocytes, we next assessed the quantitative levels of fibrocytes in the bone marrow, circulation (i.e., buffy coat cells), and lungs of these animals, as compared to controls. Fibrocytes were markedly expanded in the bone marrow, increased in the circulation, and increased in the lungs of NY1DD mice (Figure 4A, 4B, and 4C, respectively). The same chemokine receptor hierarchy was found on fibrocytes from the bone marrow, circulation, and lungs, CXCR4≫CCR2>CCR7 (Figure 4A, 4B, and 4C, respectively). To further confirm that these were fibrocytes and not macrophages that had phagocytized collagen, FACS analysis was performed on these same cells to detect intracellular pro-collagen type I and III by using specific antibodies that detect the C-terminus and N-terminus of type I pro-collagen and the C-terminus of pro-collagen III. CD45+ pro-collagen type I and III positive cells were expanded in the bone marrow, increased in the circulation and lungs of NY1DD mice (Figure 4D, 4E, and 4F, respectively). In addition, we found increased numbers of fibrocytes that appeared to be undergoing differentiation to αSMA+ cells in the bone marrow, circulation, and lungs of NY1DD SCD mice (Figure 4G, 4H, and 4I, respectively).

### NY1DD SCD mice displayed increased numbers of fibrocytes in their bone marrow, circulation, and lungs under conditions simulating VOC (hypoxia followed by reoxygenation)

**Figure 5:**
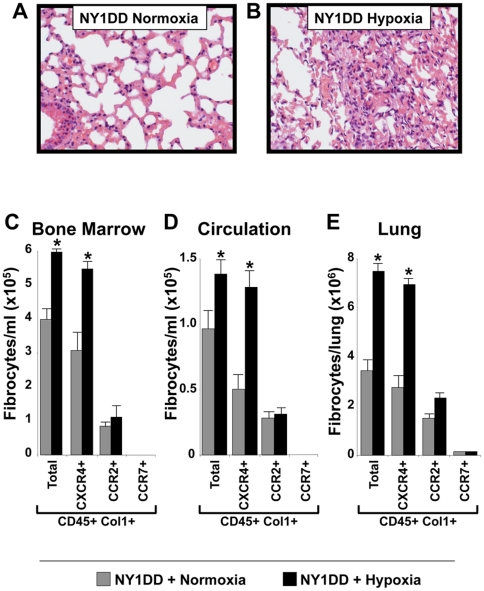
NY1DD SCD mice displayed marked lung fibrosis and inflammation under conditions simulating VOC (hypoxia followed by reoxygenation) (B), as compared to NY1DD mice exposed to normoxic conditions alone (A). Photomicrographs are representative H&E of lungs from six mice in each group, under 400×. NY1DD SCD mice displayed increased numbers of fibrocytes in their bone marrow, circulation, and lungs under conditions simulating VOC (hypoxia followed by reoxygenation). C–E) demonstrates that fibrocytes are elevated in the bone marrow, circulation, and lungs of NY1DD SCD mice under conditions simulating VOC (hypoxia followed by reoxygenation), as compared to NY1DD mice exposed to normoxic conditions alone. In addition, C–E) demonstrates that fibrocytes in the bone marrow, circulation, and lungs of NY1DD SCD mice under conditions simulating VOC (hypoxia followed by reoxygenation), as compared to NY1DD mice exposed to normoxic conditions alone express a chemokine receptor hierarchy (i.e., CXCR4+≫CCR2+>CCR7+). N = six mice in each group. * p = <0.05.

To determine whether NY1DD SCD mice experience worsening of their chronic ILD with hypoxia followed by reoxygenation (normoxia) similar to patients with SCD that experience VOC, we placed NY1DD SCD mice and wild type (C57BL/6) mice in hypoxia (8% oxygen) for 3 hrs followed by return to normoxia for 4 hrs using a modification as previously described [31]. Hypoxia followed by reoxygenation in NY1DD SCD mice resulted in marked worsening histopathology of the lungs of NY1DD SCD (Figure 5A and 5B), as compared to normoxic-exposed mice, consisting of areas of fibrosis and inflammation. These findings recapitulated what we had found in our previous studies using this mouse model of SCD [31]. To determine whether the marked change in pathology under conditions of hypoxia/reoxygenation was associated with a change in the number of fibrocytes, we measured fibrocytes under the above conditions in the bone marrow, circulation, and lungs of these mice. We found a marked increase in numbers of fibrocytes in the bone marrow, circulation, and lungs under conditions of hypoxia followed by normoxia, as compared to NY1DD SCD mice exposed only to normoxia or to control mice (Figure 5C, 5D, and 5E, respectively). The chemokine receptor hierarchy that was found expressed on fibrocytes under these conditions in the bone marrow, circulation, and lungs demonstrated the persistent expression pattern of CXCR4≫CCR2>CCR7 (Figure 5C, 5D, and 5E, respectively).

### NY1DD SCD mice have increased lung levels of CXCL12 and other cytokines that are relevant to fibrocyte biology

**Figure 6:**
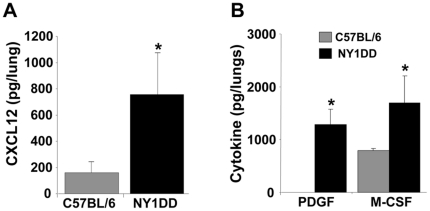
NY1DD SCD mice have increased lung levels of CXCL12 and other cytokines that are relevant to fibrocyte biology. A) demonstrates that the ligand to CXCR4, CXCL12, is markedly elevated in the lungs of NY1DD mice under baseline conditions compared to appropriate strain and age-match mice. B) demonstrates that the growth factor (PDGF) and colony stimulating factor (M-CSF) are significantly elevated in the lungs of NY1DD mice under baseline conditions compared to appropriate strain and age-match mice. N = six mice lungs in each group. * p = <0.05.

On the basis of the presence of increased fibrocytes expressing CXCR4 as the predominant chemokine receptor in the lungs of NY1DD mice under either baseline or hypoxia/normoxic conditions, we next determined whether CXCL12, the putative ligand to CXCR4, was elevated in the lungs of NY1DD mice under baseline conditions. We found that CXCL12 in lung homogenates of NY1DD mice is markedly elevated and similar to the levels of CXCL12 that we had found in bleomycin exposed lungs on days 8 to 20, which also correlated with maximal extracellular matrix deposition in this model (Figure 6A) [13]. Immunohistochemistry for CXCL12 in the lungs of NY1DD mice demonstrated that CXCL12 was localized to a variety of cells in the lung parenchyma that included type II pneumocytes. Moreover, when we measured other cytokines thought to be relevant to fibrocyte biology [34], [35], we found elevated levels of PDGF and M-CSF in the lungs of these animals (Figure 6B).

### Depletion of CXCL12 under baseline conditions in NY1DD SCD mice reduces the number of fibrocytes, and this is correlated with a reduction in collagen deposition and improvement in lung compliance and histopathology

**Figure 7:**
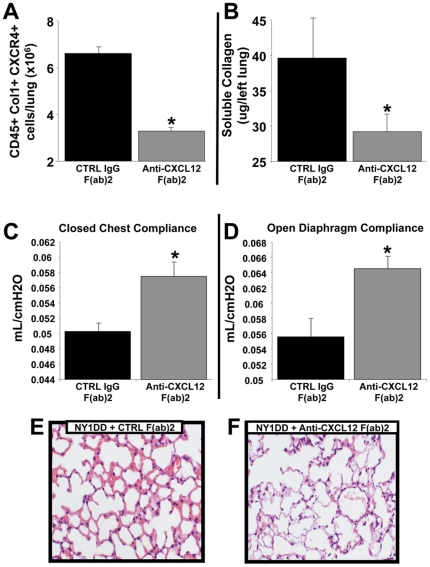
Depletion of CXCL12 under baseline conditions in NY1DD SCD mice reduces the number of fibrocytes within their lungs, and this is correlated with a reduction in collagen deposition and improvement in lung compliance and histopathology. A) demonstrates that passive immunization of NY1DD mice with anti-CXCL12, as compared to appropriate control F(ab)2 antibodies markedly attenuates the presence of fibrocytes in the lungs of NY1DD mice under baseline conditions. B) demonstrates that passive immunization of NY1DD mice with anti-CXCL12, as compared to appropriate control F(ab)2 antibodies reduces the amount of soluble collagen in the lungs of NY1DD mice under baseline conditions. C–D) demonstrates that passive immunization of NY1DD mice with anti-CXCL12, as compared to appropriate control F(ab)2 antibodies improves the compliance of the lungs of NY1DD mice under baseline conditions. E–F) demonstrates that passive immunization of NY1DD mice with anti-CXCL12 (F), as compared to appropriate control F(ab)2 antibodies (E) improves the histopathology of the lungs as demonstrated by the H&E photomicrographs. N = six mice in each group for A–D, lung photomicrographs (E–F) are representative of six lungs from each group. * p = <0.05.

On the basis of the presence of CXCL12 in the lungs of NY1DD mice under homeostatic conditions that directly correlated with the presence of increased numbers of CXCR4+ fibrocytes, we next examined whether depletion of CXCL12 would impact on extravasation of fibrocytes into the lungs of NY1DD mice, and whether this would have a direct effect on the magnitude of collagen deposition. NY1DD mice under baseline conditions were treated with neutralizing anti-CXCL12 or control antibodies (i.p. Q48 hrs) for a period of 7 days at which point they were sacrificed and their lungs assessed for levels of fibrocytes by quantitative FACS and soluble collagen by the Sircol assay as previously described [13], [30]. We found a marked attenuation of CD45+Col1+CXCR4+ fibrocytes in the lungs of the NY1DD mice that had been depleted of CXCL12 (Figure 7A), which directly correlated with a reduction in soluble collagen (Figure 7B). Moreover, when we assessed the impact of this therapeutic strategy on other populations of leukocytes in the lungs of these mice, we found no significant impact on CD4, CD8, NK cells, neutrophils, or macrophages (Table 2). These findings suggest that the lungs of NY1DD mice are undergoing collagen deposition under baseline conditions that appears to be related to extravasation of CXCR4+ fibrocytes into their lungs. In conjunction with these studies we measured lung compliance (i.e., using FlexiVent) under both conditions of closed chest and open diaphragm and found that blocking CXCL12 resulted in improved lung compliance (Figure 7C and 7D) that was associated with markedly less ILD histopathology (Figure 7E and 7F).

**Table 2:** Passive immunization of NY1DD mice with anti-CXCL12, as compared to appropriate control F(ab)2 antibodies does not alter the extravasation of populations of other leukocytes into the lungs of these mice under baseline conditions.

		Positive cells/Right Lung (×10^5^)	
		Anti-CXCL12 F(ab)2	Control IgG F(ab)2
CD3 T-cells	CD3+	17.2+/−2.3	19.8+/−0.62
CD4 T-cells	CD3+ CD4+	6.6+/−0.61	8.2+/−0.99
CD8 T-cells	CD3+ CD8+	7.1+/−1.1	8.6+/−0.59
NK cells	CD3+ NK1.1+	6.1+/−0.31	9.5+/−1.4
Neutrophils	CD11b+ Ly6g+	21.4+/−1.5	28.3+/−2.1
Macrophages	MAC3+CD11b-CD11c+	23.3+/−2.5	26.6+/−4.2

## Discussion

This study provides the first evidence that circulating CD45+Col I+CXCR4+ fibrocytes, recruited through the CXCL12/CXCR4 axis, contribute to the expansion of the fibroblast/myofibroblast-like cell population in people and mice with SCD. At baseline, NY1DD mice have increased fibrocytes in the bone marrow, circulation and lungs compared to controls and, similar to studies of subjects with IPF, the fibrocytes express the hierarchy of chemokine expression CXCR4≫CCR2>CCR7. The CXCR4 ligand, CXCL12, is critical to CXCR4+ fibrocyte trafficking and neutralizing CXCL12 prevents fibrocyte extravasation into the lungs. These findings in NY1DD mice complement our initial findings in adult patients with SCD providing preliminary evidence for a role for fibrocytes in the development of human ILD.

The pathogenesis of ILD in patients with SCD is poorly understood. Previously, ILD in this patient population was thought to be solely the result of repetitive infarcts occurring during ACS episodes [2]. Recently, a new paradigm has emerged for the understanding of vascular occlusion and end-organ damage in SCD, suggesting that SCD should be viewed as a disease characterized by subclinical, ongoing microvascular occlusion and punctuated by clinically apparent exacerbations [31], [36], [37]. In this model of SCD pathogenesis, end organ damage in SCD results from the total injury that occurs from clinical exacerbations plus ongoing, subclinical events. The findings of this study demonstrating organ damage and elevated levels of circulating fibrocytes at baseline in NY1DD mice support this notion of chronic microvascular occlusion, systemic inflammation and ongoing injury and repetitive repair, and provide a novel mechanism for the understanding of lung fibroproliferation in chronic ILD in patients with SCD.

Since the initial observations of ILD in patients with SCD were published [2], newer data describing the role of nitric oxide depletion, hemolysis and pulmonary hypertension in pathogenesis of SCD has emerged [4], [5]. In a prior study, Powars et al describe chronic sickle cell lung disease as a disorder characterized by pulmonary fibrosis, pulmonary hypertension and a mean survival of 5 years [2]. More recently, several large studies have demonstrated an increased risk of death associated with pulmonary hypertension in adults with SCD [5], [38], [39]. Potentially, the mortality risk associated with chronic sickle cell lung disease in the prior study was due to pulmonary hypertension and not pulmonary fibrosis. However, examining the etiology of ILD in patients with SCD is important, because ILD has been shown to contribute to the development of pulmonary hypertension in SCD [40]. Although the findings in current study implicate fibrocytes in the pathogenesis of ILD, future studies should focus on the potential role of fibrocytes in the vascular remodeling of pulmonary hypertension in SCD.

Circulating fibrocytes contribute to the fibroproliferation in the lung seen in NY1DD mice. At baseline, there is increased collagen deposition in the lung of NY1DD mice compared to control mice demonstrating the active fibroproliferation in the lungs of NY1DD mice. In the bone marrow, circulation and lungs of NY1DD mice, fibrocytes are increased compared to controls and there is a subpopulation of αSMA+ cells, consistent with a fibroblast/myofibroblast-like cell. Although we cannot define the lineage of αSMA+ cells based on our data, the presence of elevated αSMA+ fibrocytes in the bone marrow of NY1DD mice suggests that these cells are beginning to undergo differentiation to a fibroblast/myofibroblast-like cells in the bone marrow. Consistent with our findings of CXCR4 predominance on circulating fibrocytes in other mouse models of pulmonary fibrosis [23], [24], the majority of fibrocytes in NY1DD mice express CXCR4. The only known ligand for CXCR4, CXCL12, was markedly elevated in the lungs of NY1DD mice compared to controls, and neutralizing CXCL12 attenuated extravasation of fibrocytes into the lungs of NY1DD mice and reduced lung collagen deposition. Taken together, these findings support the notion that, at baseline, fibrocytes are expanded in the bone marrow, mobilized to the circulation, home and extravasate in the lung of SCD mice dependent upon the CXCR4/CXCL12 chemokine axis.

In NY1DD mice, fibrocytes increase during hypoxia/reoxygenation compared to levels during normoxic conditions. Although no animal model fully recapitulates human disease, inducing hypoxia in NY1DD mice is thought to be analogous to a VOC in patients with SCD [31], [36]. Given that the natural history of SCD is characterized by disease exacerbations or VOC [41], hypoxia/reoxygenation in the mouse model approximates human disease. The current data suggest that VOC causes increased fibrocyte mobilization into circulation in both human SCD and the mouse model, and potentially promotes increased lung fibrosis.

Similar to findings in the NY1DD mouse, fibrocytes are elevated at baseline in patients with SCD compared to healthy control subjects. The chemokine hierarchy of CXCR4≫CCR2>CCR7 was also present in patients with SCD, suggesting that the CXCR4/CXCL12 chemokine axis is used for fibrocyte trafficking. Although circulating CXCL12 was higher in patients with SCD compared to controls, this did not reach statistical significance. CXCL12 was only measured in a subset of our patients and this may have limited our ability to detect a difference between cases and controls. Moreover, the lung should be the site for elevated levels of CXCL12 in order to promote a “chemotactic gradient” for CXCR4+ fibrocytes; and we found elevated levels of CXCL12 in the lungs of NY1DD mice. However, since plasma levels of CXCL12 were not significantly different between SCD subjects and controls, we can only postulate that in human SCD the CXCR4/CXCL12 chemokine axis and the gradient of CXCL12 in the lung is responsible for fibrocyte homing. During a VOC, fibrocyte levels within SCD subjects increased significantly over baseline and demonstrated an activated phenotype. Repeat measurements within an individual using a longitudinal study design provides assurance that the findings are not due to inter-individual variation. Our study was not designed to determine cause and effect between VOC and fibrocyte levels in patients with SCD. We can only report an association. Regardless of whether vaso-occlusion causes increased fibrocyte levels or vice versa, higher levels of fibrocytes in association with VOC may promote fibroproliferation and end organ damage. These data are consistent with NY1DD mice and support the notion that disease exacerbations (i.e., VOC) significantly contribute to the development of end organ disease. Furthermore, these data provide preliminary evidence that the pathophysiology and pathology we found in the NY1DD mouse can be translated to patients with SCD.

Taken together, our data in NY1DD mice and supported by evidence in patients with SCD suggests a novel model of ILD pathogenesis in SCD. This new paradigm for understanding the mechanism of pulmonary fibrosis in SCD implicating bone-marrow-derived fibrocytes is consistent with findings in IPF. Based on the data from this study, we postulate that inflammatory “signals” (i.e., CXCL12) generated in the lung communicate with the bone marrow leading to expansion of fibrocytes. The condition of vaso-occlusion (i.e., either subclinical or VOC) in the bone marrow creates an environment that is hypoxic in nature, which favors the induction of CXCR4 expression on fibrocytes that ultimately enhances their homing and extravasation at the target organ site (i.e., lung) in response to CXCL12. These circulating fibrocytes, largely CXCR4+, will only home and extravasate into an end-organ (i.e., lung) if there is the appropriate “address” signal, such as lung-specific expression of a chemokine ligand (i.e., CXCL12) to CXCR4. Once fibrocytes home and extravasate into the lung dependent on the CXCL12/CXCR4 biological axis, the data from our NY1DD mice suggests that they play a major role in functioning as mesenchymal progenitor cells for the production of extra-cellular matrix and contribution to fibrosis in the lungs of patients with SCD.

In summary, our results provide a new basis for understanding the pathogenesis of ILD in patients with SCD, a common and serious complication in this patient population [2], [3], [6]. The findings of our study offer the opportunity to therapeutically target fibrocytes by attenuating their recruitment into the lung in order to prevent pulmonary fibrosis.. Future studies are needed to examine strategies to attenuate fibrocytes and determine the impact on the longitudinal development of ILD in patients with SCD.

## Supporting Information

Supplemental Information S1 Detailed Methods section.
(DOC)
