# Supplementary material for: The Role of Fibrocytes in Sickle Cell Lung Disease
Source: PLoS One. 2012 Mar 19;7(3):e33702. doi: 10.1371/journal.pone.0033702 (PMC3307761; doi:10.1371/journal.pone.0033702)
Supplement: Supplemental Information S1 — Detailed Methods section. (DOC) [file pone.0033702.s001.doc]

*Supplemental Information S1*

**The Role of Fibrocytes in Sickle Cell Lung Disease**

Joshua J. Field, Marie D. Burdick, Michael R. DeBaun, Brett A. Strieter, Ling Liu,

Borna Mehrad, C. Edward Rose Jr., Joel Linden, and Robert M. Strieter

**METHODS**

Human subjects

Blood samples were obtained from persons with SCD, ages 19 years and older, as well as race-matched controls. Samples were taken during a routine office visit to the Adult Hemoglobinopathy Clinic at Washington University in St. Louis from persons with SCD who reported no pain or no more than typical pain. SCD diagnoses were confirmed by hemoglobin analysis. In a subset of SCD subjects, additional blood samples were collected during a hospitalization for a VOC. The definition of VOC was pain in the extremities, back, abdomen, chest, or head for which no other explanation other than SCD could be found [1]. Additionally, the primary diagnosis for the hospital admission was required to be VOC per the attending hematologist. The subset of patients with paired baseline and pain samples represents a convenience sample of subjects who presented to the hospital with pain. These samples were collected without regard to prior morbidities. Race-matched African-American controls were healthy employees of Washington University, who were also appropriately consented for study participation. The healthy controls did not have a history of respiratory disease, inflammatory conditions or current smoking.

The Institutional Review Board (IRB) at Washington University and the University of Virginia approved all human protocols and written informed consent was obtained in accordance with the Declaration of Helsinki. IRB approval numbers are PRO00014330 and 15598.

Animal model of sickle cell disease

Experiments were performed on an established breeding colony of twelve to sixteen week old transgenic sickle cell mice on a C57BL/6 genetic background described as NY1DD mice [2,3,4,5]. The animal studies were carried out in accordance with the recommendations in the Guide for the Care and Use of Laboratory Animals of the National Institutes of Health and were approved by the Committee on the Ethics of Animal Experiments of the University of Virginia (Permit Number: 3549).

Sircol assay

The Sircol assay (Biocolor Ltd., Belfast, United Kingdom) was performed using the manufacturer’s instructions [6]. Briefly, the mouse lungs were homogenized in 1 ml of complete lysis buffer (Roche, Indianapolis, IN). 50 ul of the lung homogenate or collagen standard was added to 50 ul of 0.5 M acetic acid and 1.0 ml of sircol dye reagent was added and mixed for 30 minutes. The collagen-dye complex was precipitated by centrifugation at 16,000 g for 10 minutes, and dissolved in 1.0 ml of 0.5 M NaOH. Finally, the samples were introduced into a microplate reader and the absorbance determined at 540 nm.

Morphometric analysis and picrosirius red staining

Paraffin-embedded sections (3 μm thick) of NY1DD lung and bleomycin exposed control lung were immersed in xylene (twice, 1 minute each) and then washed initially in 100% ethanol (twice, 1 minute), followed by 50% ethanol (twice, 1 minute), and finally rinsed in distilled water for 10 minutes. Subsequently, the sections were treated with 0.2% molybdophosphoric acid (2 minutes), rinsed in distilled water (2 minutes), and then exposed to a 0.1% solution of picrosirius red stain for 90 minutes. After picrosirius red staining, the sections were again rinsed in distilled water (1 minute) and then 50% ethanol (twice, 1 minute each), 100% ethanol (twice, 1 minute each), and finally xylene (1 minute). Next, ten fields per section of random lung specimens were morphometrically assessed for the area of picrosirius red staining. For quantitative assessment of the areas of positive picrosirius red staining, we used NIH 1.55 imaging software [7].

Fibrocyte, cytokine/chemokine measurement

Fibrocytes, cytokine and chemokine measurements were performed from the circulation, bone marrow and lungs in NY1DD mice. Quantitative FACS analysis for fibrocytes (defined as CD45+Col1+; further confirmed as CD45+ type I and III pro-collagen+ cells), alpha smooth muscle actin (αSMA)+ cells (CD45+αSMA+Col1+, TGF-β activated fibrocytes (CD45+Col1+pSmad2/3+) fibrocytes expressing chemokines receptors (CXCR4, CXCR7, CCR2, CCR7, CCR5, and in combination), αSMA+ cells expressing chemokine receptors (CXCR4, CXCR7, CCR2, CCR7, CCR5, and in combination), and leukocytes (CD3, CD4, CD8, NK (NK1.1+CD3-), Neutrophils (CD11b+Ly6g+), and Macrophages (MAC3+CD11b-CD11c+)) using a modification as described [8,9,10]. All antibodies were purchased from BD Biosciences except aSMA (R&D Systems, Minneapolis, MN), Col1 (Rockland, Gilbertsville, PA), and the Pro-collagen antibodies (Millipore, Billerica, MA). Plasma from retro-orbital or right ventricle (RV) blood samples were analyzed for cytokines by Luminex multiplex protein analysis using a modification as previously described [9]. Isolated buffy-coat cells were processed for complete blood count, differential, and quantitative FACS analysis for fibrocytes, αSMA+ cells, TGF-β activated fibrocytes, fibrocytes expressing chemokines receptors, αSMA+ cells expressing chemokine receptors, and leukocytes as described above for bone marrow using a modification as described [8,9,10]. For human SCD samples, peripheral blood (5ml) was collected at Washington University in St. Louis, MO, and shipped overnight on wet ice to the laboratory (RMS) in Charlottesville, VA. The same protocols used for fibrocyte, chemokine/cytokine measurements in NY1DD mice as described above were also used for human samples. Note: the number and activation state of fibrocytes in blood on wet ice is stable for 48 hrs (data not shown).

Hypoxia/reoxygenation, analogous to vaso-occlusion

The protocol for hypoxia/reoxygenation was similar to previous studies [11,12,13,14,15,16]. NY1DD Mice were exposed to either ambient oxygen (21% oxygen/79% nitrogen) or hypoxia (8% oxygen/92% nitrogen) for 3 hours followed by reoxygenation to room air (21% oxygen/79% nitrogen) for 4 hours followed by sacrifice. Fibrocyte numbers (with and without expression of specific chemokine receptors) and activated fibrocyte phenotype (i.e., pSmad2/3+ and αSMA+) were then measured at time of sacrifice in three compartments, bone marrow, blood, and lungs from these SCD and control mice.

Depletion of CXCL12

Preparation of anti-CXCL12 antibody was performed as previously described [9,17]. In experiments where NY1DD mice were depleted of CXCL12, mice were passively immunized with anti-CXCL12 antibody for 10 days prior to sacrifice using a modification as previously described [9,17].

Lung mechanics

Lung mechanics of the animals were assessed by FlexiVent using Quasi-static pressure-volume (PV) curve to calculate total lung compliance and elastance, and forced oscillatory measurements to measure frequency dependence of parenchymal tissue impedance and parenchymal tissue elastance according to manufacturer’s instructions.

Statistical analysis

For human subjects, fibrocyte levels were not normally distributed. To assess differences in fibrocyte levels between cases and controls, Mann-Whitney U tests were used. To determine if differences were present in paired measurements (baseline vs. VOC), Wilcoxon matched-pairs signed-ranks tests were used For mouse studies, fibrocyte data were normally distributed and comparisons between two groups of mice were performed with Student’s t tests. Probability values were considered statistically significant if they were less than 0.05. Data analysis was performed in SAS version 9.1 (SAS Institute, Cary, NC).

**REFERENCES**

1. Platt OS, Thorington BD, Brambilla DJ, Milner PF, Rosse WF, et al. (1991) Pain in sickle cell disease. Rates and risk factors. N Engl J Med 325: 11-16.

2. Fabry ME, Costantini F, Pachnis A, Suzuka SM, Bank N, et al. (1992) High expression of human beta S- and alpha-globins in transgenic mice: erythrocyte abnormalities, organ damage, and the effect of hypoxia. Proceedings of the National Academy of Sciences of the United States of America 89: 12155-12159.

3. Fabry ME, Nagel RL, Pachnis A, Suzuka SM, Costantini F (1992) High expression of human beta S- and alpha-globins in transgenic mice: hemoglobin composition and hematological consequences. Proc Natl Acad Sci U S A 89: 12150-12154.

4. Dasgupta T, Hebbel RP, Kaul DK (2006) Protective effect of arginine on oxidative stress in transgenic sickle mouse models. Free Radic Biol Med 41: 1771-1780.

5. Lunzer MM, Yekkirala A, Hebbel RP, Portoghese PS (2007) Naloxone acts as a potent analgesic in transgenic mouse models of sickle cell anemia. Proc Natl Acad Sci U S A 104: 6061-6065.

6. Blease K, Schuh JM, Jakubzick C, Lukacs NW, Kunkel SL, et al. (2002) Stat6-deficient mice develop airway hyperresponsiveness and peribronchial fibrosis during chronic fungal asthma. Am J Pathol 160: 481-490.

7. Keane MP, Belperio JA, Arenberg DA, Burdick MD, Xu ZJ, et al. (1999) IFN-gamma-inducible protein-10 attenuates bleomycin-induced pulmonary fibrosis via inhibition of angiogenesis. J Immunol 163: 5686-5692.

8. Gomperts BN, Strieter RM (2007) Fibrocytes in lung disease. J Leukoc Biol 82: 449-456.

9. Phillips RJ, Burdick MD, Hong K, Lutz MA, Murray LA, et al. (2004) Circulating fibrocytes traffic to the lungs in response to CXCL12 and mediate fibrosis. J Clin Invest 114: 438-446.

10. Mehrad B, Burdick MD, Zisman DA, Keane MP, Belperio JA, et al. (2007) Circulating peripheral blood fibrocytes in human fibrotic interstitial lung disease. Biochem Biophys Res Commun 353: 104-108.

11. De Franceschi L, Platt OS, Malpeli G, Janin A, Scarpa A, et al. (2008) Protective effects of phosphodiesterase-4 (PDE-4) inhibition in the early phase of pulmonary arterial hypertension in transgenic sickle cell mice. FASEB J 22: 1849-1860.

12. de Franceschi L, Baron A, Scarpa A, Adrie C, Janin A, et al. (2003) Inhaled nitric oxide protects transgenic SAD mice from sickle cell disease-specific lung injury induced by hypoxia/reoxygenation. Blood 102: 1087-1096.

13. Sabaa N, de Franceschi L, Bonnin P, Castier Y, Malpeli G, et al. (2008) Endothelin receptor antagonism prevents hypoxia-induced mortality and morbidity in a mouse model of sickle-cell disease. J Clin Invest 118: 1924-1933.

14. Belcher JD, Mahaseth H, Welch TE, Vilback AE, Sonbol KM, et al. (2005) Critical role of endothelial cell activation in hypoxia-induced vasoocclusion in transgenic sickle mice. Am J Physiol Heart Circ Physiol 288: H2715-2725.

15. Osarogiagbon UR, Choong S, Belcher JD, Vercellotti GM, Paller MS, et al. (2000) Reperfusion injury pathophysiology in sickle transgenic mice. Blood 96: 314-320.

16. Wallace KL, Marshall MA, Ramos SI, Lannigan JA, Field JJ, et al. (2009) NKT cells mediate pulmonary inflammation and dysfunction in murine sickle cell disease through production of IFN-gamma and CXCR3 chemokines. Blood 114: 667-676.

17. Phillips RJ, Burdick MD, Lutz M, Belperio JA, Keane MP, et al. (2003) The stromal derived factor-1/CXCL12-CXC chemokine receptor 4 biological axis in non-small cell lung cancer metastases. Am J Respir Crit Care Med 167: 1676-1686.
